# Cognition Improvement in Taekwondo Novices Over 40. Results from the SEKWONDO Study

**DOI:** 10.3389/fnagi.2013.00074

**Published:** 2013-11-11

**Authors:** Gaby Pons van Dijk, Marjolein Huijts, Jan Lodder

**Affiliations:** ^1^Department of Neurology, School for Mental Health and Neuroscience, University Hospital Maastricht, Maastricht, Netherlands; ^2^Cardiovascular Research Institute Maastricht, University of Maastricht, Maastricht, Netherlands

**Keywords:** Taekwondo, senior citizens, cognition, hard martial arts, volunteers over 40

## Abstract

Age-related cognitive decline is associated with increased risk of disability, dementia, and death. Recent studies suggest improvement in cognitive speed, attention, and executive functioning with physical activity. However, whether such improvements are activity specific is unclear. Therefore, we aimed to study the effect of 1 year age-adapted Taekwondo training on several cognitive functions, including reaction/motor time, information processing speed, and working and executive memory, in 24 healthy volunteers over 40. Reaction and motor time decreased with 41.2 and 18.4 s (*p* = 0.004, *p* = 0.015), respectively. Digit symbol coding task improved with a mean of 3.7 digits (*p* = 0.017). Digit span, letter fluency, and trail making test task-completion-time all improved, but not statistically significant. The questionnaire reported “better” reaction time in 10 and “unchanged” in 9 of the 19 study compliers. In conclusion, our data suggest that age-adapted Taekwondo training improves various aspects of cognitive function in people over 40, which may, therefore, offer a cheap, safe, and enjoyable way to mitigate age-related cognitive decline.

## Introduction

Age-related cognitive decline is associated with increased risk of disability, dementia, and death. Especially in Western societies this problem is expected to increase in the coming decades, underscoring the need for strategies to reduce the chance of cognitive decline (Alzheimer’s Association, 2009). Recent studies suggest improvements on cognitive speed, auditory and visual attention (Angevaren et al., 2008), and executive functioning (Colcombe and Kramer, 2003) by increasing physical activity. The cardiovascular fitness hypothesis of McAuley et al. proposes that the effect of physical activity on cognitive function is mediated by two mechanisms: (1) disease reduction (e.g., stroke, diabetes, hypertension, and cardiovascular disease), and (2) enhancement in brain structure and function (e.g., increased production and efficiency of neurotransmitters, angiogenesis, synaptogenesis, and neurogenesis) (McAuley et al., 2004). Changes in aerobic fitness are thus suggested to precede changes in cognitive function (Angevaren et al., 2008). Contemporary dance (Coubard et al., 2011) and bio-energetic sports (Colcombe and Kramer, 2003; McAuley et al., 2004; Angevaren et al., 2008) have been shown to improve several aspects of cognition in the elderly. Practicing Tai Chi (Toskovic et al., 2004; Man et al., 2010; Lam et al., 2011), a “soft” martial art, had similar effects. We recently showed that age-adapted Taekwondo training is feasible, safe, and fun, whereas it improved the overall feeling of wellbeing (Pons van Dijk et al., 2013a). Since Taekwondo training improves aerobic performance capacity, it could also improve cognitive function, or at least mitigate age-related cognitive decline (Wolter and Studenski, 1996). In the absence of any study so far, we aimed to study the effect of 1 year Taekwondo training on several cognitive functions, including reaction time (RT) and motor time (MT), information processing speed, and working and executive semantic memory, in a group of healthy, volunteers over 40. If effective, such Taekwondo-based exercise program could offer a cheap method to lower age-related cognitive decline, which eventually might contribute to mitigate age-related loss of self-maintenance in the general population.

## Materials and Methods

Study methods as to participant recruitment, sample size estimation, and intervention were described in detail elsewhere (Pons van Dijk et al., 2013b). We listed only those items relevant to the present study. For the study protocol description, see the internet link below. The study was designed as a single arm intervention study in which 24 participants served as their own control.

### Study population

Twelve male and 12 female volunteers between 41 and 71 years of age, see Table 1.

**Table 1:** **Participants demographics**.

Nr	Gender	Age	Education level	Physical activity (h/week)
1	M	61	U	15
2	M	61	U	10
3	M	62	U	0
4	M	59	U	7
5	F	41	U	0
6	M	58	U	9
7	M	51	U	4
8	F	49	H	3
9	F	60	U	10
10	M	66	O	10
11	F	50	H	5
12	M	57	U	4
13	F	51	H	3
14	F	63	U	0
15	M	70	U	2
16	M	53	U	5
17	F	50	H	5
18	M	52	H	3
19	F	57	H	4
20	M	59	U	0
21	F	60	U	1
22	F	44	H	10
23	F	51	H	1
24	F	48	U	0

*U, master degree or equivalent; H, bachelor degree or equivalent*.

*O, other; Gray, non-complier*.

### Inclusion criteria

Willingness to follow at least a 1 h Taekwondo training session weekly. All volunteers had a routine neurological and cardiologic physical investigation and an exercise ECG.

### Exclusion criteria

Current psychiatric treatment, the use of oral anticoagulants or any disease that was expected to interfere with training, as judged by the volunteer’s treating physician, the study neurologist (JL), or study cardiologist.

### Recruitment

Posters inviting people for participation were posted at various locations in the Maastricht University Medical Center and the Maastricht University. People interested in the study were informed by oral and written information, and they were encouraged to follow at least two Taekwondo sessions at the trainers’ Taekwondo club before enrollment.

### Ethical consideration

The study was conducted according to the principles of the Declaration of Helsinki, and in accordance with the Dutch Medical Research Involving Human Subjects Act (WMO). It was approved by the Maastricht University Medical Center Ethical Committee and listed before study onset in the linked, Dutch Trial Registry: www.trialregister.nl/trialreg/admin/rctsearch.asp under the acronym “SEKWONDO Study.” Informed consent was provided and signed before initiation of the study.

#### Intervention

The weekly training sessions lasted about 1 h. Training intensity was adjusted to the participants’ physical condition, and adapted over time. Training sessions started on 21 October, 2009, and ended on 19 January, 2011. We aimed at 40 lessons for each participant in 1 year, but since attendance rate was lower than anticipated, we decided to extend the training period with 3 months to a total of 15 months. The training sessions lasted 1 h, and generally consisted of the following elements: a warming-up with muscle strengthening and light stretching exercises; Taekwondo techniques, such as style figures (poomsae), which are fixed patterns illustrating a fight against one or more imaginary opponents; stances, blockings, kicks, and punches, which were trained as basic exercises, with kicking pads, or facing an opponent. A small number of elementary self-defense techniques based on Taekwondo elements were also included. Training sessions ended with a cooling-down, including stretching exercises (http://www.wtf.org/) (Lodder, 2012). To prevent possible injuries, Taekwondo combat sparring was not practiced. For more details see Pons van Dijk et al. (2013a,b).

#### Measurements

All participants were tested 1 month before the start of the study, and in the month after the last training session.

### Reaction test

This test consists of two parts. In the first part participants are asked to hold their index finger on a golden button. As soon as a yellow light appears on the screen they should press a black button as quickly as possible, and then return to the golden button as quickly as possible. In the second part, participants are asked only to respond (by pressing the black button) when the yellow light appears on the screen together with hearing a tone. Other conditions like a red light or a yellow light with no tone were “no go’s,” and participants should withhold their response. This task measures RT and motor speed. RT is the time from signal to the moment of releasing the golden button. The MT is the time from releasing the golden button to pressing the black button (Schufried, 2001). The numbers 1 and 2 refer to the part for which the RT and MT were measured.

### Digit symbol coding [Wechsler Adult Intelligence Scale fourth edition (WAIS-IV)]

This test is based on the substitution of numbers into symbols according to an explicit code. It provides information on motor speed, information processing speed, and visuo-motor coordination. The raw score corresponds to the number of symbols substituted correctly within 120 s (Wechsler, 2001).

### Digit span (WAIS-IV)

A series of numbers, starting with three consecutive numbers and a maximum of eight consecutive numbers, is being read to the participant. In the first subtask (forward), the participant is asked to repeat the number series. In the second subtask (backward), the participant is asked to repeat the numbers, but in reverse order. This test assesses short memory and working memory, respectively (Wechsler, 2001).

### Letter fluency

Participants are asked to name as many words as possible starting with a certain letter within a certain time frame. This task provides information on the ability to retrieve semantic memory items in a strategic way (Lezak et al., 2004).

### Trail making test A and B

In the first subtask (card A), the numbers 1–13 are placed in random order on a sheet of paper, and participants are asked to connect the numbers in ascending order as fast as possible. In the second subtask (card B) numbers and letters are placed in random order, which have to be connected alternately in ascending and alphabetical order. An interference score was computed as time on card B minus time card A. This task provides information on the ability to concentrate, set-shifting, visual perception/attention, working memory, and the ability to concentrate (Reitan, 1956).

### Questionnaire

A questionnaire on the participants’ subjective assessment on various aspects of the program was conducted at the end of the training period. There were two questions relevant to cognitive function:

Since I started practicing Taekwondo training: My mental fitness is:/ My reaction time is: (answer options: *much worse/worse/unchanged/better/much better*).

### Statistical analysis

Data were analyzed using SPSS, version 17.0, SPSS Inc., Chicago, IL, USA. Baseline and final group data were compared using non-parametric Wilcoxon Signed-Rank Test and paired *t*-test, expressed as mean with standard deviation (SD). A *p*-value < 0.05 was considered statistically significant.

## Results

Median participants’ ages was 57 (range: 41–71) years. All participants had baseline and final measurements, so there were no study dropouts. However, three women and two men withdrew early from the training program (non-compliers). Demographic characteristics of the non-compliers did not differ from those of study compliers (Table 1).

**Figure 1:**
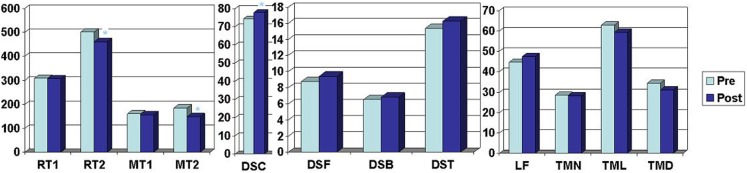
**Outcome measurements compilers**. RT1, reaction time light; RT2, reaction time light and sound; MT1, motor time light; MT2, motor time light and sound; DSC, digit symbol coding; DSF, digit span forward; DSB, digit span backward; DST, digit span total; LF, letter fluency; TMN, trail making numbers; TML, trail making letters; TMD, trail making difference.

Results of pretest and posttest measurements for the 19 compliers are shown in Table 2 and Figure 1.

**Table 2:** **Outcome measurements compliers**.

Measurement	Pre	SD	Post	SD	Significance
**REACTION TIME(S)**					
Light	310.0	55.7	307.4	50.1	0.784
Light and sound	502.5	95.1	461.4	76.2	0.004
**MOTOR TIME(S)**					
Light	161.9	58.8	157.0	58.0	0.359
Light and sound	168.1	74.3	149.7	58.6	0.015
**DIGIT SYMBOL CODING (#)**					
Symbols substituted	74.2	10.8	77.8	12.6	0.017
**DIGIT SPAN (#)**					
Forward	8.79	1.75	9.42	1.80	0.083
Backward	6.53	1.74	6.84	1.38	0.542
Total	15.37	3.02	16.26	2.92	0.120
**LETTER FLUENCY (#)**					
Total words	44.47	10.7	47.42	10.5	0.064
**TRAIL MAKING(S)**					
Numbers	28.42	10.1	28.26	9.07	0.939
Letters	62.89	20.0	59.26	15.7	0.143
Difference	34.47	15.0	31.00	13.5	0.304

*Mean, # is number*.

### The reaction test

RT and MT measured in the first task decreased (non-significantly) with 2.5 and 4.9 s, respectively. RT and MT in the second subtask decreased with 41.2 and 18.4 s, respectively; both differences being statistically significantly. Nine participants showed lower values (median 36 s, range 5–96) in RT1, and 16 participants (median 45 s, range 9–180) in RT2. Ten participants had higher RT1 values (median 21 s, range 5–53), and three higher RT2 values (median 59 s, range 3–71). The MT1 decreased in 12 participants with a median of 22 s (range 3–48), and MT2 in 13 with a median of 18 s (range 4–115). Seven participants had higher MT1 values (median 19 s, range 9–36), and seven higher MT2 values (median 7.5 s, range 1–22).

### The digit symbol coding task

The digit symbol coding task values decreased significantly with a mean of 3.7 digits (*p* = 0.017). Fourteen participants had lower values with a median of six digits (range 1–14), and five participants had higher values with a median of four digits (range 3–5).

### The digit span

The digit span increased, forward with 0.6 digits, backward with 0.3 digits, and the total digit span increased with 0.9 digits; these changes were not statistically significant. An increase of digit span was seen in nine participants with a median of 1 digit (range 0–3), eight participants did not change, whereas two participants had lower values with a median of 1.5 digits (range 1–2) in the digit span forward. In the backward digit span eight participants had higher values with a median of 1.5 digits (range 0–3), seven participants showed no change, whereas four participants had lower values with a median of 2 digits (range 1–4). Sixteen participants showed higher values with a median of 2 digits (range 1–4), and three had lower values with a median of 2 digits (range 1–4).

### The letter fluency test

Overall there was a mean increase of three letters (*p* = 0.06). Fourteen participants showed an increase with a median of eight letters (range 1–14), and five participants a decrease with a median of six letters (range 2–13).

### The trail making test

The task-completion-time decreased in the first part with a mean of 0.16 s, the second part with 3.6 s, whereas the difference decreased (not statistically significant) with 3.5 s. The first part showed a decrease in nine participants with a median of 6 s (range 1–20), whereas 10 participants showed an increase with a median of 4 s (range 1–20). The second part showed a decrease in 14 participants with a median of 16.5 s (range 2–28), and an increase in five participants with a median of 18 s (range 9–31). Regarding the difference between the two tasks, 11 participants decreased with a median of 11 s (range 6–48), while 8 participants increased with 9.5 s (range 1–34).

### The questionnaire

Five of the 19 compliers reported “better” mental fitness, whereas 14 reported “unchanged.” Ten of the 19 study compliers reported “better” RT, whereas nine reported this item as “unchanged.” All non-compliers reported “unchanged” on both items.

## Discussion

Our study showed that a weekly 1 h Taekwondo training for the duration of 15 months improves several aspects of cognition in participants over 40, in particular cognitive speed relating to inhibition improved, as well as in information processing.

One part of Taekwondo is sparring, in which apart from tactics, speed and technique are important elements. We included various sparring-related techniques in our program (although never with actual physical contact) which focused on acquiring controlled motor speed and reflex motor reaction time, as well as timely inhibition of movements (Martinez and Caraway, 1982). Other training aspects, which involve RT and inhibition, were punching and kicking exercises using pads, in which one has to master motor control and motor speed. Such exercises may also stimulate visuo-motor coordination.

We found no other studies on the effects of Taekwondo on cognitive functions in people over 40. One recent study in 15 male participants practicing karate, a hard martial arts resembling Taekwondo, found no improvement in RT1 reaction time (Chateau-Degat et al., 2010; Pons van Dijk et al., under submission).

Recent studies on aerobic exercise, like swimming and running, showed improvements in cognitive functioning in elderly people (Colcombe and Kramer, 2003; Smith et al., 2010). However, many community-dwelling elderly do not exercise because of little physical strength and multiple medical conditions (Chang et al., 2011). Therefore, exercises should be adapted to their physical condition, like our program of age-adapted Taekwondo (Pons van Dijk et al., 2013a). A relationship between training intensity and degree of results by physical exercise is highly plausible. However, the difficulty is that training intensity is hard to define as opposed to training frequency or duration when it concerns complex movement patterns as opposed to the training of less complex character, such as running, e.g., However, the characterization of the movement complexity may well reflect the degree of training intensity to eventually master them. We cannot describe the complexity of Taekwondo poomsae execution, but this may be appreciated by observing such execution at for example: www.wtf.org.

As mentioned above we also found an improvement in information processing, combined with motor speed and visuo-motor coordination. Especially processing speed is one of the cognition components which deteriorates with age (Salthouse, 1996; Albinet et al., 2012) and is demanded in more complex tasks during daily activities, such as for example walking and talking at the same time. The other components on which our participants improved, mainly consist of tasks related to working memory and executive functions.

The improvements we found did not relate to improvement of cardiac function (exercise ECG data not reported here), which suggests that exercise-related improvement of cognitive functions do not solely depend on cardio-aerobic improvement. We suspect that cognitive improvement in our study resulted from the training of rather complex motor patterns which are inherent to Taekwondo, especially during style or poomsae training. The participants practiced three different poomsaes, which demanded memory training for complex motor sequences, as well as mental concentration and attention. Poomsae performance resembles Tai Chi, with the difference that poomsae training is much more dynamic in terms of alteration of initiation, performance, and inhibition of complex movement patters. Several studies demonstrated improved cognitive function by Tai Chi practice (Chan et al., 2005; Lam et al., 2009). Because of the more dynamic character of Taekwondo RT and MT, as well as working memory and information processing speed may be stimulated even more than by Tai Chi training, although this cannot be established with certainty without direct comparing both arts.

Our study has limitations, one of which is the absence of a control group. However, as we did not expect performances to improve with another year of aging, participants functioned as their own controls. One may question the specificity of the intervention on study outcome parameters, as most participants were also physically active in other ways. However, first of all other activities included also mild exercising such as walking, whereas we aimed to indicate that our population was not completely sedentary. Furthermore, we considered the Taekwondo training as the intervention on top of the participants’ habitual activities, and considered responsible for the effects we found. However, without a strict controlled design we cannot be fully certain of the intervention’s specificity. Another limitation concerns extrapolating our results to people over 40 in general, since our sample was rather small, whereas our participants were on average highly educated. The early withdrawal of five participants could be regarded as a study drawback, but these five still completed follow-up measurements, and are “non-compliers” and not study dropouts. Moreover, based on our sample size estimation for the primary outcome measure, which was a balance parameter, eventual complete study adherence in 17 would suffice.

Due to an increase of the number of elderly people, cognitive impairment is becoming a major societal problem. There are still few, if any, solutions to this problem. Pharmaco-therapeutical possibilities are just emerging but so far had only minor impact on a population level, whereas as it offers no certainty for any improvement on an individual level (Tariot et al., 2004; Kaduszkiewicz et al., 2005). Other options, for instance day care and other entertaining programs put high demands on money and personal. Our results suggest that Taekwondo-based exercises may offer a way to mitigate age-related cognitive decline, which, moreover, is cheap, safe, and enjoyable (Pons van Dijk et al., 2013a).

In perspective, our results show that practicing Taekwondo improves various aspects of cognitive function in people over 40. Earlier we found that such practice is cheap, safe, and enjoyable (Pons van Dijk et al., 2013a) whereas it also improves balance maintenance (Pons van Dijk et al., 2013b). The practice does not require exhaustive training intensity to achieve results. Considering the already available training infra-structure by means of the wide spread presence of Taekwondo clubs in many countries worldwide, from the perspective of health improvement on a societal level governmental policy may already be directed toward offering this training possibility to the general public. However, more studies on the effects of Taekwondo on cognitive function in the elderly are required to make specific recommendations as to when to begin such training and with what degree of intensity it should be practiced for optimal results. Besides, Taekwondo organizations and their trainers should be made aware of this new and important possibility Taekwondo has to offer.

## Conflict of Interest Statement

The authors declare that the research was conducted in the absence of any commercial or financial relationships that could be construed as a potential conflict of interest.
